# The role of small RNAs on phenotypes in reciprocal hybrids between *Solanum lycopersicum* and *S. pimpinellifolium*

**DOI:** 10.1186/s12870-014-0296-1

**Published:** 2014-11-01

**Authors:** Junxing Li, Qian Sun, Ningning Yu, Jiajin Zhu, Xiaoxia Zou, Zhenyu Qi, Muhammad Awais Ghani, Liping Chen

**Affiliations:** Institute of Vegetable Science, Zhejiang University, 866 Yuhangtang Road, Hangzhou, Zhejiang Province P.R. China; Key Laboratory of Horticultural Plants Growth, Development and Biotechnology, Agricultural Ministry of China, Hangzhou, 310058 P.R. China; Fuli Institute for Food Science, College of Biosystems Engineering and Food Science, Zhejiang University, Hangzhou, 310058 P.R. China

**Keywords:** Tomato, Reciprocal hybrids, Phenotypic variation, Small RNAs

## Abstract

**Background:**

Reciprocal hybrids showing different phenotypes have been well documented in previous studies, and many factors accounting for different phenotypes have been extensively investigated. However, less is known about whether the profiles of small RNAs differ between reciprocal hybrids and how these small RNAs affect gene expression and phenotypes. To better understand this mechanism, the role of small RNAs on phenotypes in reciprocal hybrids was analysed.

**Results:**

Reciprocal hybrids between *Solanum lycopersicum* cv. Micro-Tom and *S. pimpinellifolium* line WVa700 were generated. Significantly different phenotypes between the reciprocal hybrids were observed, including fruit shape index, single fruit weight and plant height. Then, through the high-throughput sequencing of small RNAs, we found that the expression levels of 76 known miRNAs were highly variable between the reciprocal hybrids. Subsequently, a total of 410 target genes were predicted to correspond with these differentially expressed miRNAs. Furthermore, gene ontology (GO) annotation indicated that those target genes are primarily involved in metabolic processes. Finally, differentially expressed miRNAs, such as miR156f and 171a, and their target genes were analysed by qRT-PCR, and their expression levels were well correlated with the different phenotypes.

**Conclusions:**

This study showed that the profiles of small RNAs differed between the reciprocal hybrids, and differentially expressed genes were also observed based on the different phenotypes. The qRT-PCR results of target genes showed that differentially expressed miRNAs negatively regulated their target genes. Moreover, the expression of target genes was well correlated with the observations of different phenotypes. These findings may aid in elucidating small RNAs contribute significantly to different phenotypes through epigenetic modification during reciprocal crossing.

**Electronic supplementary material:**

The online version of this article (doi:10.1186/s12870-014-0296-1) contains supplementary material, which is available to authorized users.

## Background

Wide hybridization is a common phenomenon in plant evolution that has made a great contribution to the improvement of crops by transferring many desired traits from wild species to crops, such as rice [1], wheat [2], and sun-flower [3]. Moreover, the significantly different phenotypes between the reciprocal hybrids have been well documented in several different plant species. For example, an earlier study using *Arabidopsis thaliana* as a maternal parent and *A. arenosa* as a paternal parent showed that many live seeds were produced, though the reciprocal hybrids could not be obtained [4]. In some cases, vigour is different between reciprocal hybrids, such as between *A. thaliana* ecotypes C24 and Col-0 [5]. Despite ample experimental evidence for the occurrence of this phenomenon, many different mechanisms, including parent-origin effects [6], dosage-sensitive regulators [7], gene imprinting [8], transposable elements activated [9], cytoplasmic-nuclear interaction [10], maternal effects [11], cytoplasmic inheritance [12-14], the dominance model [15], overdominant effects and epistasis [16-19], have been proposed to understand the different phenotypes between reciprocal hybrids.

Previous studies have shown that epigenetic modifications, especially those involving small RNAs, are a main factor for the development and growth of plants. Therefore, we speculate the intriguing possibility that epigenetic modifications may play an important role in different phenotypes between reciprocal hybrids. Small RNAs including miRNAs and siRNAs, which function as mediators and regulators, play an extensive role in epigenetic processes and gene expression. For example, 24-nt siRNAs can mediate DNA methylation and the silencing of transposons [20-22], and 21-nt siRNAs and miRNAs can regulate the gene expression levels through cleaving target genes [23,24]. According to previous studies, hybridization may induce changes in small RNAs [25-28]. In addition, Li [29] found that the change in small RNAs by grafting (asexual hybridization) could result in the phenotypic variations. However, less is known about what happens to epigenetics between the reciprocal hybrids, and how epigenetics may affect the gene expression and phenotypes of reciprocal hybrids. Therefore, finding the differences in small RNAs after hybridization and how these small RNAs regulate gene expression and subsequent phenotypes between reciprocal hybrids is worth exploring.

Tomato is a model plant and a very important economic vegetable crop [30]. Wild tomatoes contain a higher nutrition quality and more disease-resistance genes and also exhibit a higher feasibility to cross with cultivated tomatoes [31,32]. Distant hybridization is usually applied to incorporate these preferable traits from wild tomatoes into the cultivars for genetic improvements. In the present study, a reciprocal cross between the cultivar and wild tomato was first established to determine whether different phenotypes between the reciprocal hybrids exist. Second, based on the different phenotypes, small RNAs were analysed by high-throughput sequencing to explore any differences between reciprocal hybrids. Third, the expression of predicted target genes corresponding to differentially expressed miRNAs was analysed by qRT-PCR to observe the correlation between genes and phenotypes. These results suggest that small RNAs may be responsible for the phenotypic variations in reciprocal hybrids.

## Results

### Phenotypic analysis of the reciprocal hybrids and their parents

**Figure 1:**
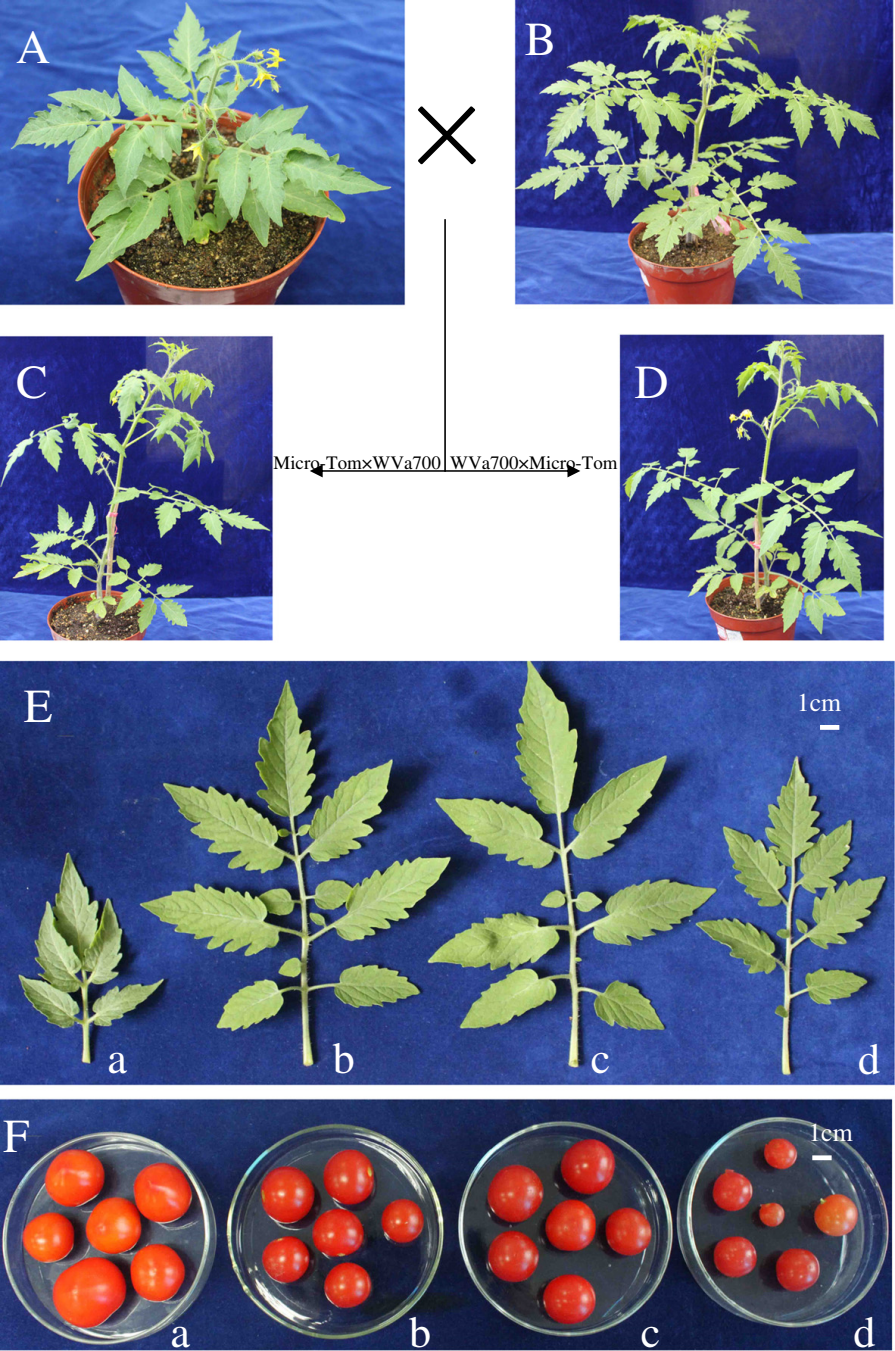
**Parents and their reciprocal hybrids: (A) Micro-Tom; (B) WVa700; (C) Micro-Tom × WVa700; (D) WVa700 × Micro-Tom; (E) the leaf of the plant: a. Micro-Tom, b. Micro-Tom × WVa700, c. WVa700 × Micro-Tom, d. WVa700; (F) the fruits of the plant: a. Micro-Tom, b. Micro-Tom × WVa700, c. WVa700 × Micro-Tom, d. WVa700.**

To find out whether there are different phenotypes between the reciprocal hybrids of the distant hybridization, the reciprocal cross between *Solanum lycopersicum* cv. Micro-Tom and *S. pimpinellifolium* line WVa700 was performed, and the phenotypic characterizations of the hybrids were analysed (Figure 1). The data showed that Micro-Tom × WVa700 had larger leaf area, crown width and smaller fruit shape index than their parents, whereas longer leaf length and smaller fruit shape index were found in WVa700 × Micro-Tom when compared with parents (Additional file 1). In addition, the results also showed that Micro-Tom × WVa700 exhibited significantly larger fruit shape index and smaller single fruit weight and plant height compared with WVa700 × Micro-Tom (Figure 1E; F; Additional file 1). Therefore, phenotypes of fruit shape index, single fruit weight and plant height were dramatically different between the reciprocal hybrids.

### Small RNAs sequencing in reciprocal hybrids and their progenitors

Mature small RNAs are generated in the cytoplasm; therefore small RNAs in reciprocal hybrids with different cytoplasms were analysed by high-throughput sequencing to determine whether there are differences between them and explore the relationship of small RNAs with gene expression and phenotypes in the reciprocal hybrids. Four separate small RNA libraries (Micro-Tom, WVa700, Micro-Tom × WVa700 and WVa700 × Micro-Tom) were generated and their sequencing data have been deposited into the SRA database of NCBI with accession number SRX722032, SRX722033, SRX722034 and SRX722035, respectively.

A total of 12657989, 11212106, 11263114 and 11227866 reads were obtained from leaf libraries of Micro-Tom, WVa700, Micro-Tom × WVa700 and WVa700 × Micro-Tom, respectively, after eliminating reads without sRNA sequences ranging from 15 to 30 nt in length (Additional file 2). The length distribution was primarily 20–24 nt, in which 21 nt and 24 nt lengths were most abundant at approximately 16% and 45%, respectively. Compared to WVa700 × Micro-Tom, 21 nt and 24 nt sRNAs in Micro-Tom × WVa700 were more abundant. Among all four types of tomatoes, the accumulation of 24 nt sRNAs was higher than that of 21 nt sRNAs.

### Analysis of the repeat-associated siRNAs

A total of 12519660, 11081459, 11125150 and 11030188 clean reads were obtained from Micro-Tom, WVa700, Micro-Tom × WVa700 and WVa700 × Micro-Tom, respectively, including miRNA, rRNA, repeat, snRNA and others (Additional file 3; Additional file 4). Note that the top four of the repeat-associate siRNAs were matched on the sequences of LTR in both the unique tags and total tags. Surprisingly, all four types of repeat-associate siRNAs accumulated to lower levels in WVa700 × Micro-Tom relative to those in Micro-Tom × WVa700 (Additional file 5; Additional file 6). siRNA is derived from repetitive sequences, mediates RNA-dependent DNA methylation and is important in gene expression. Thus, the differences in abundance of repeat-associate siRNA may influence the chromatin stability and gene expression of reciprocal hybrids.

### Analysis of known miRNAs between reciprocal hybrids

Known miRNAs were found by the miRBase tool. After searching the sequences, 44 conserved miRNAs belonging to 25 families were detected (Additional file 7). Moreover, the abundance of each family was analysed (Additional file 8). A dramatic difference was found between the abundances of different families. The reads of four families (miR157, miR166, miR167 and miR168) were significantly higher than those of other families. Interestingly, compared with WVa700 × Micro-Tom, the abundance of miRNAs in the four families of Micro-Tom × WVa700 were higher, indicating that the miRNAs of the four families may be fundamental and indispensable factors for plant growth and development in tomato and may contribute to the different gene expressions between the reciprocal hybrids.

**Figure 2:**
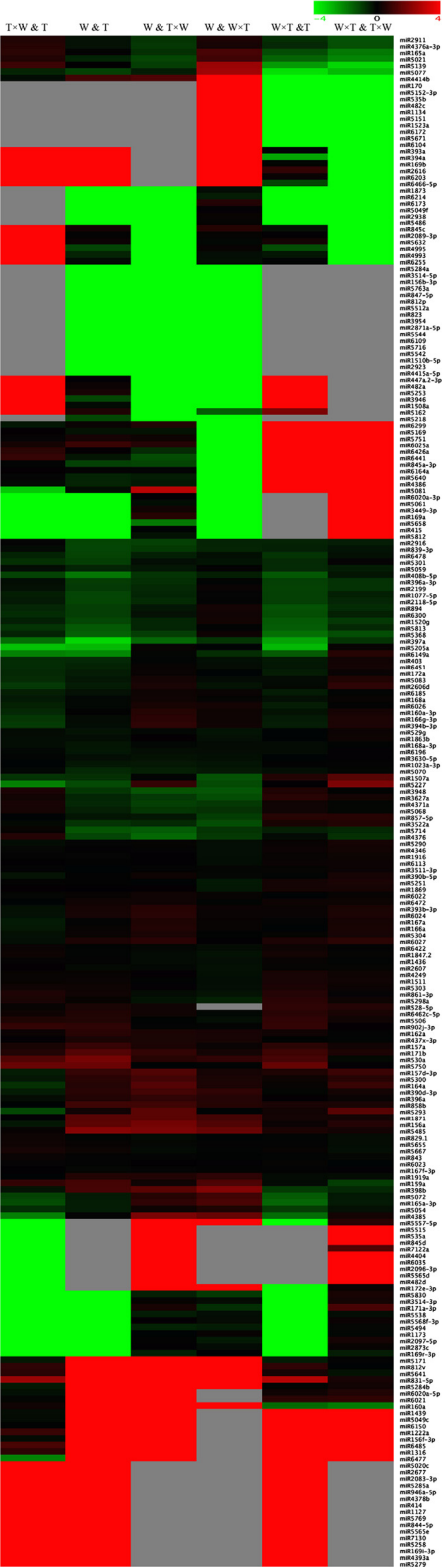
**The different expression of miRNAs in the leaves between the reciprocal hybrids and the parents displayed with hierarchical cluster analysis.**

To explore the different influences of miRNAs on phenotypes between reciprocal hybrids, differentially expressed known miRNAs were analysed by the approach of hierarchical cluster (Figure 2). The expression levels of 76 miRNAs including 13 conserved and 63 non-conserved miRNAs were highly variable between the reciprocal hybrids, and a total of 63 miRNAs displayed a greater than four-fold change (Additional file 9). Among them, the expression of 40 miRNAs in Micro-Tom × WVa700 were higher than those of WVa700 × Micro-Tom, such as conserved miRNAs (miR156f-3p, miR171a-3p, miR535a and miR169a) and non-conserved miR5081 that showed similar expression levels between Micro-Tom and WVa700. The expression levels of the other 36 miRNAs, including miR482c, miR394a, miR535b, miR169b, miR170, miR393a, miR160a and miR165a, were obviously lower in Micro-Tom × WVa700. Hence, the differentially expressed miRNAs may be relevant to significantly different phenotypes between reciprocal hybrids.

**Figure 3:**
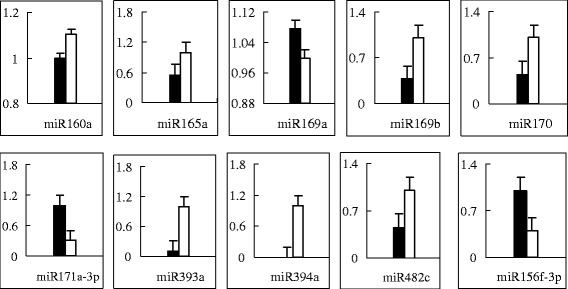
**The validation of differently expressed miRNAs in reciprocal hybrids.** Black pillars represent miRNAs of Micro-Tom × WVa700 and white pillars represent miRNAs of WVa700 × Micro-Tom.

To validate the different levels of miRNA expression, 10 conserved miRNAs were tested in quantitative experiments by stem-loop RT-PCR. The results of the quantitative experiments were consistent with the sequencing data (Figure 3).

### The prediction of target genes of differently expressed miRNAs

The target genes of differently expressed miRNAs were predicted to elucidate the relationship between miRNAs and phenotypes.

**Figure 4:**
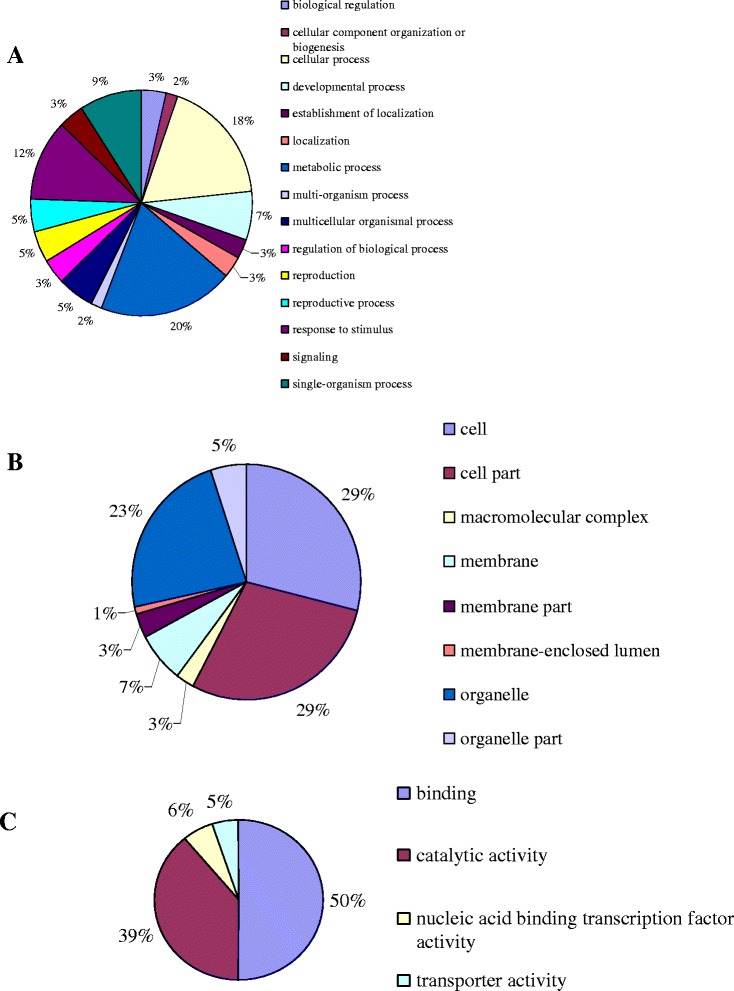
**The GO (Gene ontology) annotation of target genes. A**. biological process, **B**. cellular component, **C**. molecular function.

A total of 410 target genes for 76 differentially expressed miRNAs were predicted. The gene functions of these targets were determined by gene ontology (GO) annotation and involved biological processes, cellular components and molecular functions (Figure 4). The top three biological processes were metabolic processes (20%), cellular processes (18%) and response to stimuli (12%). Moreover, those target genes were primarily located within the cell, cell parts and organelles at 29%, 29% and 23%, respectively. In addition, 50% of target genes for molecular function were attributed to binding and 39% were attributed to catalytic activity, indicating that those targets may be involved in many metabolic processes and that there may be complicated relationships between those targets and different phenotypes.

**Figure 5:**
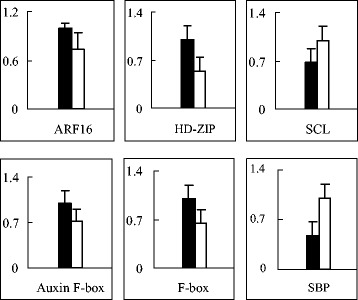
**The expression of the target genes of differentially expressed miRNAs in reciprocal hybrids.** Black pillars represent target genes of Micro-Tom × WVa700 and white pillars represent those of WVa700 × Micro-Tom.

To interpret the possible specific relationships of the targets and different phenotypes between the reciprocal hybrids, the quantitative RT-PCR analysis was used to measure the expression levels of six predicted target genes that are involved in the development of leaves, including *ARF16* (miR160a), *HD-ZIP* (miR165a), *Auxin F-box protein* (miR393a), and *F-box protein* (miR394a) [33-36], the development of fruits, including *SBP* (miR156f-3p) [37], and plant height, including *SCL* (miR171a-3p) [38] (Figure 5). The results showed that the expression levels of *SBP* and *SCL* were higher in WVa700 × Micro-Tom than those of Micro-Tom × WVa700, whereas *ARF16*, *HD-ZIP*, *Auxin F-box protein* and *F-box protein* were lower in WVa700 × Micro-Tom. Therefore, the expression levels of target genes were negatively correlated with the abundances of their corresponding miRNAs in this study.

## Discussion

Different phenotypes in reciprocal hybrids have been well documented in several different plant species. In the present study, a significantly larger fruit shape index and smaller single fruit weight and plant height was found in Micro-Tom × WVa700 compared with WVa700 × Micro-Tom. Therefore, understanding how different phenotypes occur after reciprocal cross is important.

### Different profiles of 24-nt sRNAs in reciprocal hybrids

miRNAs are often 21 nt or 22 nt in length, whereas siRNAs are 24 nt length [39]. In the present study, the top two abundant sRNAs were miRNAs (approximately 16%) and siRNAs (approximately 45%) as determined by high-throughput sequencing, which is similar to a previous study on the tomato plant that showed that 24-nt sRNAs accumulated more than 21-nt sRNAs [40].

From the length distribution of sRNAs, 24-nt sRNAs were present in the highest proportion of the total sRNAs, ranging from 47.51% (Micro-Tom × WVa700) to 42.62% (WVa700 × Micro-Tom) (Additional file 2), and the trend was consistent with the total DNA methylation levels in reciprocal hybrids. The results also showed that the total DNA methylation levels in Micro-Tom × WVa700 were insignificantly higher than that of WVa700 × Micro-Tom (unpublished results). Hence, the different profiles of 24-nt sRNAs may influence the expression of associated genes to regulate the phenotypes. Furthermore, among the top four repeat-associate siRNAs, all matched to an LTR (a type of retrotransposon) that had higher levels in Micro-Tom × WVa700 than those of WVa700 × Micro-Tom (Additional file 5 and Additional file 6). Moreover, the LTR can be reactivated by interspecific hybridization, which has been demonstrated in several previous studies [41,42]. Therefore, we deduced that the different reactivity of LTR regulated by different profiles of repeat-associate siRNAs may influence the phenotypic variation between reciprocal hybrids.

### Different phenotypes may be caused by differently expressed miRNAs

Previous studies have reported that gene regulation through sequence specific interactions between miRNAs and their target genes can affect plant growth and development. In a previous study, the loss-of-function mutant of *ARF16* (*MIR160a* gene) was used to find intriguing phenotypes in the leaf [33], suggesting that different expression levels may influence the development of the leaf. Moreover, by targeting *HD-Zip*, Auxin F-box proteins, F-box protein genes, and miRNAs, including miR165a, miR393a and miR394a, also regulate the development of the leaf and make a contribution to the construction of leaf morphology [34-36]. In this study, the significantly different phenotypes of leaf area and leaf length between the hybrids and the parents were displayed. Meanwhile, the expressions of miR160a, miR165a, miR393a and miR394a showed dramatically different profile between the reciprocal hybrids. In addition, the fruit of Micro-Tom × WVa700 had less single fruit weight (Additional file 1), whereas miR156f-3p had significantly higher levels of expression in Micro-Tom × WVa700 compared with those of WVa700 × Micro-Tom (Additional file 9). One possibility is that the increased accumulation of miR156 led to a decrease in the expression of *SBP* that influenced fruit weight, which was confirmed in transgenic tomato plants [37]. Furthermore, *SCARECROW-LIKEA* (*SCL*), which is the target of miR171, was involved in plant height [38]. A significantly different plant height and the expression level of miR171a-3p were found in this study. In summary, the expression levels of miRNAs and target genes in reciprocal hybrids differ with different phenotypes. Therefore, the expression of miRNAs that negatively regulate their targets may contribute to different phenotypes between reciprocal hybrids during distant hybridization.

In conclusion, the primary feature of reciprocal hybrids is that they have same nuclear genomes, but their cytoplasm and epigenomes may be quite different. Attributing the different phenotypes between reciprocal hybrids solely to one factor does not aid in understanding the underlying possible molecular mechanisms behind these differences. In the present study, small RNAs including miRNAs and siRNAs exhibited differences between reciprocal hybrids. Accounting for the different patterns of mature small RNAs between reciprocal hybrids, the different modifications of MIRNA genes may be the cause of these different phenotypes due to the different epigenomes. In the cytoplasm, the single mature miRNAs are loaded into the RNA induced silencing complex to guide mRNA cleavage [39,43]. In addition, in a previous study, Lu et al. reported that maternal siRNAs can regulate the seed size in reciprocal crosses [6]. Therefore, the different cytoplasm from different maternal parents may also influence the effects of small RNAs on regulating the development of plant. In summary, further research is needed to gain a better understanding of how different profiles of small RNAs occur in reciprocal hybrids.

## Conclusions

This study showed that the profiles of small RNAs differed between the reciprocal hybrids, and differentially expressed genes were also observed based on the different phenotypes. The qRT-PCR results of target genes showed that differentially expressed miRNAs negatively regulated their target genes. Moreover, the expression of target genes was well correlated with the observations of different phenotypes. These findings may aid in elucidating small RNAs contribute significantly to different phenotypes through epigenetic modification during reciprocal crossing.

## Methods

### Plant material

*Solanum lycopersicum* cv. Micro-Tom (2n = 24) and *S. pimpinellifolium* line WVa700 (2n = 24), both pure and inbred lines, were used. Micro-Tom × WVa700 and WVa700 × Micro-Tom were obtained by crossing Micro-Tom and WVa700, respectively. Four types of 100 tomato plants, with a mean of 25 plants per type, were raised in a greenhouse at 23°C with a light/dark-period of 16-h light and 8-h dark with 60% relative humidity.

### Phenotypic characterization

Three healthy plants of the individual reciprocal hybrids, Micro-Tom, and WVa700, were randomly selected. Twenty different morphological phenotypes were observed. Leaf phenotypes were determined according to these factors, including leaf area [44], leaf length (defined as the distance from the leaf insertion point at the stem to the tip of the terminal leaflet) [45], leaf width (defined as the distance between the tips of the two longest lateral leaflets) [45], L/W of maximum leaf and the number of leaves per plant. The plant morphologies, including plant height, crown width and stem diameter, were evaluated. Leaf phenotypes and the plant morphologies of the four types of tomato plant were observed at the same stage of plant development before flowering (approximately 45 days). Moreover, some indicators of floral traits, including first flower node, number of inflorescence, flower number per inflorescence and flowering stage, were recorded. Floral traits of four types were observed at the flowering stage. In addition, the fruit traits that were studied included single fruit weight, diameter, and height; fruit shape index (h/d ratio) and in the breaker stage [46]; fruit number per inflorescence; fruiting stage; maturity stage; and fruit setting rate. Fruit traits of four types were observed at the fruit maturity. The data are the mean of three measurements and were subjected to analysis of variance (ANOVA) [47].

### High-throughput sequencing of small RNAs

While observing leaf phenotypes, three healthy plants of Micro-Tom, WVa700, Micro-Tom × WVa700 and WVa700 × Micro-Tom were also randomly selected. Total RNAs of young leaves were extracted using the Trizol reagent (Invitrogen Inc.) according to the manufacturer’s protocol. The RNAs were sent to the Beijing Genomics Institute (BGI) for sequencing. After the raw data were analysed, the clean sequences were obtained for further analyses according to the described method [48]. The clean reads were analysed by length distribution and common sequences. Then, the sequences were matched against the genome to discover the repeat associate sRNAs and to observe the expression of sRNAs and known miRNAs using the miRBase. To reveal the differential expression of miRNAs, the abundances of miRNAs in all libraries were normalized. The formula of the normalization is actual count/total count*1,000,000. Then, the values of normalization were compared between the two libraries and were calculated in the form of the fold-change (fold-change = log_2_ (treatment/control)). Moreover, the *p-value* was obtained using the formula previously described [49]. The cluster picture was generated based on the expression mode of miRNAs; in other words, the same expression mode of miRNAs would be clustered together according to their fold-change values. Regarding the prediction of target genes, the previously described rules were used [50,51]. For the prediction of targets, the gene function, including the biological process in which they involved, cellular component they located and molecular function of the genes, were analysed. The comparisons and analysis were performed between the reciprocal hybrids as well as the F_1_ hybrids (Micro-Tom × WVa700 and WVa700 × Micro-Tom) and their parents (Micro-Tom and WVa700).

### The q RT-PCR experiments

Stem-loop q RT-PCR was used for the quantification of the significantly different expressions of miRNAs. The sequences of 10 miRNAs came from the high-throughput sequencing. The primers were designed using primer software. Two micrograms of total RNA, which came from the high-throughput sRNA sequencing experiment, was converted to cDNA on the basis of the complementary designed primers.

Meanwhile, poly (A)-tailed q RT-PCR was used for the quantification of the expression of targets. The forward and reserves primers were designed by the GenScript. Two micrograms of total RNA was converted to cDNA using oligo (dT) primers.

A total of 25 μl containing 12.5 μl volumes of SYBR, 2.0 μl volumes of cDNA, 1.0 μl of forward primer, 1.0 μl of reverse primer and 8.5 μl of sterilized distilled water was amplified in a ABI STEPONE Real-Time PCR instrument. The cycling process was 95°C for 30 s, followed by 40 cycles of 5 s at 95°C and 30 s at 60°C. All reactions were performed in triplicate, and the controls with no template and no reverse transcription were performed for each gene. The threshold cycle (*C*_T_) values were obtained automatically by ABI STEPONE, and the fold changes for each gene were counted as relative quantity (RQ) values by the comparative *C*_T_ (2^-ΔΔCt^). The U6 gene and 18 s rRNA were the references for the quantification of miRNAs and their target genes, respectively. The primers are shown in the Additional file 10.

### Availability of supporting data

The supporting data of this article are included within the article and its additional files.

## Additional files

Additional file 1:
**Comparisons of phenotypic characterizations of the reciprocal hybrids and their parents.** Different letters indicate significant difference (P < 0.05). T × W, Micro-Tom × WVa700; W × T, WVa700 × Micro-Tom. Additional file 2:
**Length distribution of sRNAs in reciprocal hybrids and their parents libraries.**
Additional file 3:
**Statistics of data cleaning of sRNAs in hybrids and parents libraries.** T × W, Micro-Tom × WVa700; W × T, WVa700 × Micro-Tom. Additional file 4:
**Composition of sRNAs in reciprocal hybrids and their parents libraries.** T × W, Micro-Tom × WVa700; W × T, WVa700 × Micro-Tom. Additional file 5:
**Summary of the unique tags of the repeated associate sRNAs matched on the genomes (the used number was above 19000).**
Additional file 6:
**Summary of the total tags of the repeated-associate siRNAs matched on the genomes (the used number was above 31000).**
Additional file 7:
**Conserved miRNAs in their families in this study.**
Additional file 8:
**The abundance of miRNAs in the conserved families in this study.**
Additional file 9:
**The standardization of different expressions of miRNAs in reciprocal hybrids and their parents.** T × W, Micro-Tom × WVa700; W × T, WVa700 × Micro-Tom. Additional file 10:
**Primers used in the quantitative experiment.**
