# Supplementary material for: The role of small RNAs on phenotypes in reciprocal hybrids between Solanum lycopersicum and S. pimpinellifolium
Source: BMC Plant Biol. 2014 Nov 1;14:296. doi: 10.1186/s12870-014-0296-1 (PMC4232637; doi:10.1186/s12870-014-0296-1)
Supplement: Additional file 1: — Comparisons of phenotypic characterizations of the reciprocal hybrids and their parents. Different letters indicate significant difference (P < 0.05). T × W, Micro-Tom × WVa700; W × T, WVa700 × Micro-Tom. [file 12870_2014_296_MOESM1_ESM.doc]

Additional file 1 Comparisons of phenotypic characterizations of the reciprocal hybrids and their parents. Note: different letters indicate significant difference (P<0.05). T×W represents Micro-Tom×WVa700, W×T represents WVa700×Micro-Tom.
