# Supplementary material for: The role of small RNAs on phenotypes in reciprocal hybrids between Solanum lycopersicum and S. pimpinellifolium
Source: BMC Plant Biol. 2014 Nov 1;14:296. doi: 10.1186/s12870-014-0296-1 (PMC4232637; doi:10.1186/s12870-014-0296-1)
Supplement: Additional file 2: — Length distribution of sRNAs in reciprocal hybrids and their parents libraries. [file 12870_2014_296_MOESM2_ESM.doc]

Additional file 2 Length distribution of sRNAs in reciprocal hybrids and their parents libraries.
